# S-Adenosylmethionine (SAM) and S-Adenosylhomocysteine (SAH) Monitoring Using Analytical Methods in Clinical Laboratory Practice: Where Are We?

**DOI:** 10.3390/biomedicines14030632

**Published:** 2026-03-11

**Authors:** Antonina Kuty, Arkadiusz Kocur, Bartosz Molasy, Małgorzata Wrzosek

**Affiliations:** 1 Department of Biochemistry and Pharmacogenomics, Medical University of Warsaw, 1 Banacha St., 02-097 Warsaw, Poland; 2 Department of Drug Chemistry, Pharmaceutical and Biomedical Analysis, Faculty of Pharmacy, Medical University of Warsaw, 1 Banacha St., 02-097 Warsaw, Poland; 3 Department of Surgical Medicine, Collegium Medicum, Jan Kochanowski University of Kielce, IX Wieków Kielc 19a, 25-516 Kielce, Poland; 4 Department of Pharmaceutical Sciences, Collegium Medicum, Jan Kochanowski University of Kielce, IX Wieków Kielc 19a, 25-516 Kielce, Poland

**Keywords:** SAM, SAH, one-carbon metabolism

## Abstract

S-adenosylmethionine (SAM) and S-adenosylhomocysteine (SAH) are essential intermediates in one-carbon metabolism and key regulators of cellular methylation capacity. Their concentrations and the SAM/SAH ratio are increasingly studied as biomarkers across metabolic, cardiovascular, neurological, and cancer-related diseases. This review outlines validated analytical methods for quantifying SAM and SAH, focusing primarily on liquid chromatography–tandem mass spectrometry (LC–MS/MS), which is considered the gold standard in both clinical and research settings. A comprehensive literature search identified studies on method development, validation, and clinical use of SAM and SAH measurements. Special attention is given to analytical challenges arising from their high polarity, structural similarity, endogenous presence, and limited stability. The review also discusses preanalytical variables, including biological matrix selection, sample handling, and storage conditions. LC–MS/MS methods are compared with alternative techniques, such as immunoassays, with respect to sensitivity, specificity, matrix effects, and clinical relevance. Additionally, the review summarizes the concentration ranges of SAM and SAH, and their ratio, in healthy and patient populations, noting current standardization limitations. Overall, the review highlights the importance of harmonized analytical protocols and matrix-specific validation to enable reliable clinical interpretation of SAM and SAH as methylation biomarkers.

## 1. The Significance of One-Carbon Metabolism and DNA Methylation in Human Health and Disease

**Figure 1:**
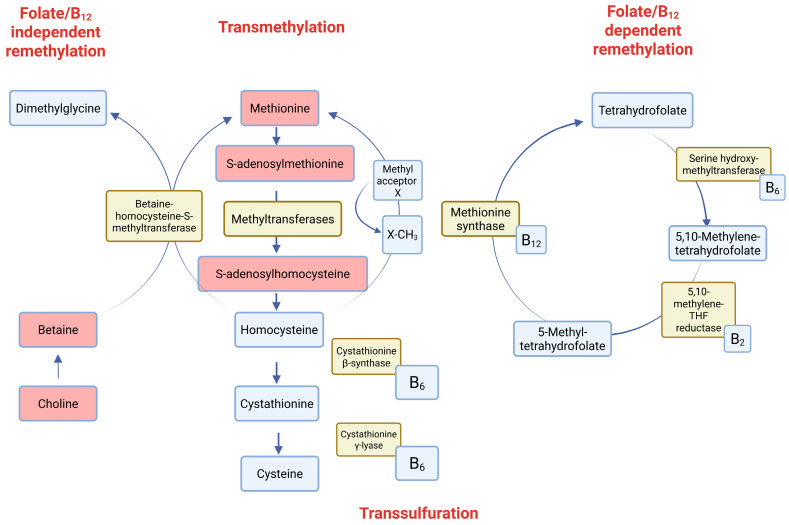
Scheme of the one-carbon group metabolism cycle. Created in BioRender. Kocur, A. (2026) https://BioRender.com/2l3bdmu. Accessed on 3 March 2026.

One-carbon metabolism is an essential biochemical pathway that integrates nutritional and genetic factors to regulate key cellular processes, including DNA methylation, nucleotide synthesis, and amino acid metabolism [1]. Through its role in epigenetic regulation, it is critical for maintaining cellular homeostasis. It involves the folate and methionine cycles, which work together to produce S-adenosylmethionine (SAM), the primary methyl donor for methylation reactions, and its by-product, S-adenosylhomocysteine (SAH), a potent inhibitor of methyltransferases [2,3,4]. The SAM/SAH ratio serves as a critical indicator of the cell’s methylation capacity. Disruptions in one-carbon metabolism—caused by dietary deficiencies of methyl-donor nutrients (such as folate, vitamins B6 and B12, choline, and betaine) or genetic variations in enzymes such as methionine synthase—can lead to imbalanced levels of SAM and SAH. These imbalances are associated with alterations in DNA methylation and the development of various health conditions, including cardiovascular diseases, neurodevelopmental disorders, mental health issues, and certain cancers. For example, vitamin B12 deficiency has been shown to lower SAM levels while increasing SAH and homocysteine, which may contribute to atherosclerosis and metabolic disturbances [5]. Furthermore, altered one-carbon metabolism has been linked to diminished cognitive function, mood disorders such as major depressive disorder, and neurodegenerative diseases such as Alzheimer’s [6,7,8,9]. During pregnancy, maternal deficiencies in methyl-donor nutrients can impair fetal development, increasing the risk of obesity, diabetes, and hypertension in offspring [10]. Recent research also indicates that one-carbon metabolites could act as biomarkers for disease severity, including in autism spectrum disorder and COVID-19 [11,12]. Because it plays a key role in connecting nutrition with gene regulation, one-carbon metabolism functions as an essential interface between environmental influences and long-term health outcomes. The scheme of pathways involved by the SAM and SAH are provided in Figure 1. Methionine is converted to S-adenosylmethionine (SAM), the principal methyl group donor, which participates in numerous methylation reactions catalyzed by methyltransferases, yielding S-adenosylhomocysteine (SAH) and subsequently homocysteine. Homocysteine may be converted to methionine via two distinct mechanisms: a folate- and vitamin B_12_-dependent pathway involving methionine synthase and 5-methyltetrahydrofolate, or a folate- and vitamin B_12_–independent route mediated by betaine derived from choline. Alternatively, homocysteine can be directed into the transsulfuration pathway, where it is metabolized to cystathionine and cysteine through vitamin B_6_-dependent enzymatic reactions. The balance of SAM-dependent methylation is tightly controlled by B-vitamin-dependent reactions, ensuring methyl group homeostasis and metabolic integrity.

Since SAM and SAH are central regulators of methyl group transfer, measuring their levels and calculating the SAM/SAH ratio is essential. Currently, chromatographic methods are regarded as the gold standard for this type of analysis; however, immunoassays (IAs) are also available for diagnostic laboratories as a more cost-effective alternative. On the other hand, LC-MS/MS (liquid chromatography–tandem mass spectrometry), due to its excellent specificity and selectivity, is a more suitable method for measuring low concentrations of SAM and SAH in the ng/mL range.

The aim of this study was to conduct a comprehensive narrative review of the current literature, focusing on LC-MS/MS-based methods for quantifying SAM and SAH. A systematic literature search was conducted in July 2025 across the PubMed, Medline, and Scopus databases, using predefined Boolean operators to ensure reproducibility. The following search syntax was applied: (“S-adenosylmethionine” OR “SAM” OR “AdoMet”) AND (“S-adenosylhomocysteine” OR “SAH” OR “AdoHcy”) AND (“LC-MS/MS” OR “liquid chromatography tandem mass spectrometry” OR “chromatography” OR “analytical method” OR “assay”).

**Figure 2:**
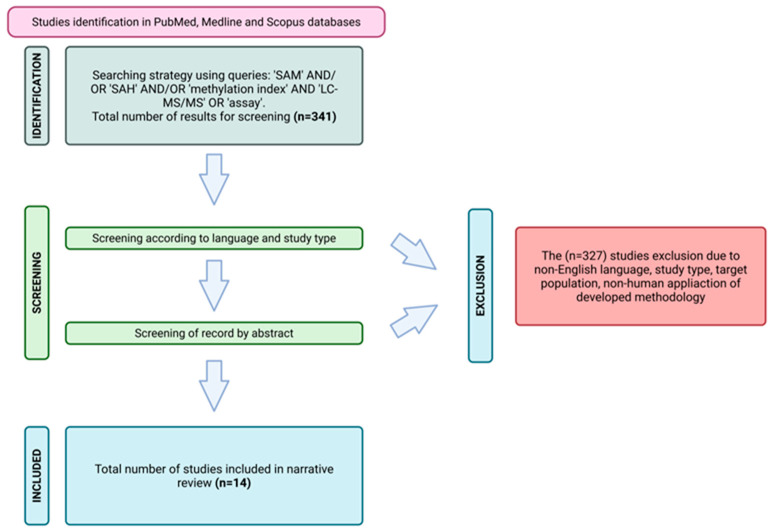
Flowchart illustrating the screening process of the manuscripts database and their inclusion/exclusion for the presented review study. Created in BioRender. Biochemii, K. (2026) https://BioRender.com/w1aqex6. Accessed on 3 March 2026.

In addition, the reference lists of selected publications were manually screened to identify further relevant studies. Articles were evaluated for relevance to the analytical determination of SAM and SAH. Duplicate records, unavailable full texts, letters to the editor, and case reports were excluded. Only English-language publications were considered. Studies lacking an analytical focus—such as those addressing exclusively clinical outcomes—or involving preclinical animal models were excluded. Following screening based on titles, abstracts, and keywords, 14 articles were included in the final review. The selection process was visualized using a PRISMA flow diagram [13] (Figure 2). Although this study is presented as a narrative review, a structured and systematic literature search was conducted to ensure the comprehensive coverage of analytical methodologies. The PRISMA flow diagram is included to transparently document the screening and selection process; however, the review does not aim to meet all criteria of a formal systematic review, such as quantitative synthesis or risk-of-bias assessment. This approach was chosen to allow methodological comparison and expert interpretation of heterogeneous analytical studies.

To the best of our knowledge, this is the first study to provide a comprehensive review of analytically validated methods for quantifying SAM and SAH using LC-MS/MS. In addition to reviewing analytical methodologies and validation strategies for SAM and SAH quantification, particular attention was paid to preanalytical stability, which is recognized as a critical factor influencing measurement accuracy. Based on the available literature, strategies for extending analyte stability were proposed. Finally, reference ranges for SAM, SAH, and the SAM/SAH ratio were summarized across the subpopulations for which data were available.

## 2. SAM and SAH—Chemical Properties as Determinants for Appropriate Analysis

**Figure 3:**
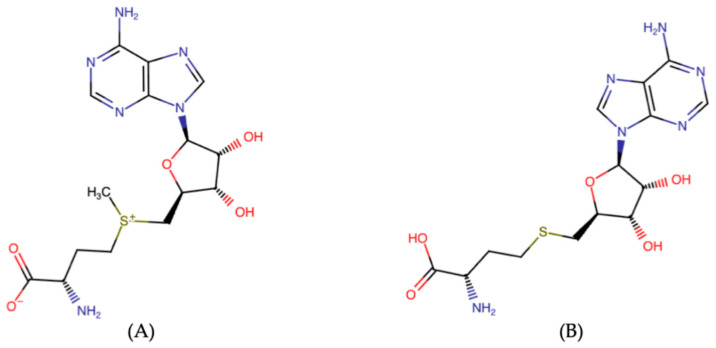
Chemical structure of SAM ((**A**); S-adenosylmethionine) and SAH ((**B**); S-adenosylhomocysteine). 2D structure image of CID 34755 (S-adenosylmethionine): PubChem Identifier: CID 34755 URL: https://pubchem.ncbi.nlm.nih.gov/compound/S-Adenosylmethionine#section=Structures. Accessed on 3 March 2026; 2D structure image of CID 439155 (S-adenosylhomocysteine): PubChem Identifier: CID 439155 URL: https://pubchem.ncbi.nlm.nih.gov/compound/S-AdenosylHOMOCYSTEINE. Accessed on 3 March 2026.

The distinct functional groups of SAM (sulfonium center) and SAH (thioether) are critical determinants for analysis. SAM is unstable, sensitive to pH, temperature, and enzymatic degradation. SAH is more stable but structurally very similar to SAM, which complicates chromatographic and mass-spectrometric separation. As presented in Figure 3 (A), the chemical structure of S-adenosylmethionine (SAM) is characterized by a positively charged sulfonium group (–S^+^–CH_3_), which makes it chemically reactive and prone to methyl transfer reactions. The presence of this labile sulfonium center influences its stability and requires careful sample handling to avoid degradation. The chemical structure of S-adenosylhomocysteine (SAH), as presented in Figure 3B, represents the demethylated product of SAM and contains a neutral thioether linkage instead of the sulfonium group. Compared to SAM, SAH exhibits higher stability. It should be emphasized that the close structural similarity between SAH and SAM necessitates highly selective analytical methods to reliably distinguish both compounds in complex biological matrices.

## 3. Preanalytical Phase—Suitable Matrix and Stability as Crucial Variables

The stability of analytes (SAM and SAH) is crucial due to the potential for hydrolysis under non-protected conditions and is strongly influenced by the type of biological matrix, storage temperature, and sample handling conditions [14,15,16]. Both molecules are highly polar, necessitating hydrophilic interaction chromatography (HILIC), ion-pairing, or LC-MS/MS approaches for reliable quantification. Various matrices, including plasma, serum, urine, and cell or organ lysates, have been considered in the published literature [5,6,7,14,17,18,19,20,21,22,23,24,25,26]. The choice of biological material depended on the study aim, as presented in Table 1. However, for diagnostic purposes, plasma/serum is generally considered the most appropriate due to its ease of collection, and it remains the most widely used and well-established biological matrix in clinical practice. In plasma, both analytes are relatively stable when samples are frozen at −80 °C, with stability confirmed for at least two weeks [5,14]. Acidification with acetic acid has been shown to improve SAM stability during longer storage [14,18,20]. Freeze–thaw cycles negatively affect recovery: %RSD values above 15% were reported, indicating that plasma samples should be analyzed immediately after thawing [7]. Nevertheless, one study demonstrated that properly acidified plasma stored at −80 °C remained stable for up to three weeks [5]. Long-term storage of prealiquoted standard stock solutions at −80 °C was reported to be stable for as long as four years [23].

In contrast to plasma, tissue extracts display marked instability. In liver and kidney samples, SAM rapidly decreased and SAH increased within minutes of incubation at 25 °C or 4 °C, resulting in a drastic drop in the SAM/SAH ratio. After only 2 min at room temperature, SAM decreased by 17%, while SAH rose by 60%, reducing the ratio by nearly half. Longer incubations (5–15 min) exacerbated these changes. Even under frozen conditions (−80 °C), prolonged storage for 2–6 months led to further decreases in SAM and increases in SAH, confirming the instability of both metabolites in tissues [17]. In embryonic tissues, SAM was shown to be unstable in plasma stored at −20 °C, while storage at −80 °C in acidified media effectively prevented degradation [20]. Cell culture models also required careful handling: frozen storage at −80 °C is generally used, but once thawed, immediate processing is essential [24].

In cerebrospinal fluid (CSF), both SAM and SAH showed good stability, with recoveries within 90–109% under frozen conditions [21]. In urine, stability data are limited; most studies normalized concentrations to creatinine, but no extended stability validation was provided [14]. For breast milk and formula samples, all fluids were stored at −80 °C until analysis, which was considered sufficient to preserve metabolite integrity [27].

In red blood cells, both SAM and SAH exhibited moderate stability when samples were collected, separated promptly, and stored at −80 °C. Stock solutions remained stable for three months at −20 ° C [26]. The stock solutions of SAM and SAH were generally stored under acidic conditions and at −80 °C to minimize degradation, particularly of SAM, which is chemically labile due to its sulfonium group. Reported data indicate that stock solutions remain stable for months to years under frozen conditions. In contrast, working solutions were typically freshly prepared or stored for limited periods, as prolonged storage may compromise analyte integrity and analytical accuracy.

The pretreatment of biological samples prior to LC–MS/MS analysis of SAM and SAH is a critical step to ensure analyte stability, remove interfering components, and achieve reliable quantification [17]. In plasma-based protocols, protein precipitation is most commonly applied after the addition of isotope-labeled internal standards [17,20,26]. Reducing agents such as DTT are frequently used [5,14,26] to prevent oxidation, followed by incubation [14,17,18,22] and centrifugation [5,6,14,17,18,19,20,21,22,23,24,25,26,27,28]. Organic solvents including acetone, acetonitrile, and methanol are widely used to precipitate proteins and lipids [29]. Alternatively, solid-phase extraction (SPE) or phenylboronic acid (PBA) cartridges were employed to selectively retain SAM and SAH via their cis-diol groups. In this approach, careful pH adjustment—initial acidification followed by neutralization—was necessary to optimize analyte binding [6]. Plasma ultrafiltration techniques combined with isotope-labeled standards have also been described [5,27]. For urine, simple dilution with water and normalization to creatinine concentrations were commonly used [14]. In cell lysates and tissue extracts protein removal was achieved by precipitation with perchloric acid, methanol, or formic acid, followed by centrifugation [17,20,22,24,25]. In one study, ultrafiltration through molecular cut-off membranes was applied to obtain a clean filtrate for analysis [23]. In red blood cell samples, trichloroacetic acid (TCA) precipitation was introduced as an additional step to ensure effective protein removal [26]. For tissues such as liver, kidney, and placenta, immediate snap freezing in liquid nitrogen after collection was critical, followed by cryogenic pulverization to prevent thawing [17,22,25]. Homogenization was performed in ice-cold perchloric acid to denature proteins and stabilize the analytes, with subsequent neutralization using potassium phosphate buffer prior to injection [17,25]. These steps were essential given the high instability of SAM and SAH in tissues at physiological temperatures. In mouse embryonic samples, homogenization was carried out in acidified mobile phase with the addition of isotope-labeled SAM, followed by heat-induced protein precipitation. This procedure enhanced throughput and reduced sample loss while maintaining analyte integrity [20]. In cerebrospinal fluid, pretreatment strategies largely mirrored those used for plasma, combining isotope-labeled internal standards, protein precipitation with organic solvents, and centrifugation [21]. For breast milk and infant formula, ultrafiltration using centrifugal membrane filters (YM-10, 10 kDa cutoff) was employed to effectively remove proteins and lipids before LC–MS/MS analysis [27]. In culture medium samples, derivatization combined with protein precipitation was applied prior to chromatographic separation [24].

## 4. Analytical Phase—LC-MS/MS as Gold Standard

Chromatographic methods, due to their specificity and sensitivity, are regarded as the gold standard in modern biochemistry, therapeutic drug monitoring, and toxicology. For complex biological matrices such as serum, plasma, and urine, these parameters are vital to ensure reliable and accurate results. Regarding SAM and SAH, the relatively low concentrations and stability issues argue against using LC-MS/MS as the preferred method. The current literature includes several studies discussing the development of LC-MS/MS methods, covering compound-related optimization and validation. In Table 1, a comprehensive summary of the studies included in the narrative review is presented.

Across published studies, presented in Table 1, concentrations of SAM and SAH measured using LC–MS/MS were most commonly quantified in plasma, but also in urine, cerebrospinal fluid, whole blood, red blood cells, tissue extracts, and cultured cell lysates. Sample volumes ranged from as low as 5–50 µL for cell and plasma samples [7,14,17,19,21,23,26] to up to 500 µL for plasma-based assays [5,6,18]. Stable isotope-labelled internal standards (mainly deuterated or 13C-labelled SAM and SAH) were consistently employed, ensuring adequate correction for matrix effects and analytical variability [5,6,14,17,18,19,20,21,22,23,26]. Unfortunately, stable isotope-labelled analogues are expensive and require a long synthesis cycle to obtain. For example, warfarin, a classic anticoagulant, has been widely used as an internal standard because of its stability and exogenous nature [30,31]. Warfarin was employed as an alternative internal standard in one of the studies from Table 1 [24], which showed that it could be eluted with a proper retention time under those chromatographic conditions. Its mass spectrum response was stable and consistent, without interference from any of the analytes [24]. In another study, described in Table 1, daptomycin dissolved in methanol was used as an internal standard, at a final concentration of 10 μg/mL [7]. Chromatographic separation was achieved using a variety of reversed-phase [21,22,26], HILIC [7], PGC [17], or amide columns [18], typically under acidic conditions with mobile phases containing formic acid [14,23,25] or acetic acid [6,24], and using gradient [5,7,18,19,20,21,22,26] or isocratic elution [14].

**Table 1:** The summary of analytical variables applied in current literature.

Analyte	Biological Matrix (Volume)	Internal Standard	Chromatography		Mass Transition (CE)	Validation Parameters		Linearity	Recovery	Reference
			Mobile Phase	Column Type		LOD	LLOQ			
SAM	Plasma (20 µL)	D_3_-SAM	0.1% formic acid in MeOH/H_2_) (5/95)	Sunfire C_8_ (3.5 µm, 4.6 × 100 mm)	399.1 → 136.1 CE = 37 V	5 nM	10 nM	10–5000 nM	~50%	[14]
	Urine (20 µL, diluted 1:25)					5 nM	10 nM	10–5000 nM	N.D.	
	Cells lysate (500,000 cells)					5 nM	10 nM	10–5000 nM	N.D.	
SAH	Plasma (20 µL)	^13^C_5_-SAH	0.1% formic acid in MeOH/H_2_ (5/95)	Sunfire C_8_ (3.5 µm, 4.6 × 100 mm)	385.1 → 136.1 CE = 23 V	1 nM	3 nM	3–5000 nM	~50%	
	Urine (20 µL, diluted 1:25)					1 nM	3 nM	3–5000 nM	N.D.	
	Cells lysate (500,000 cells)					1 nM	3 nM	3–5000 nM	N.D.	
SAM	Plasma (500 µL)	D_3_-SAM	A: 0.1 % acetic acid in H_2_O B: ACN + 0.1 % acetic acid	Atlantis T3 (3 µm, 3.0 × 150 mm)	399.1 → 136.1 CE = 25 V	2.5 nM	6.3 nM	6.3–502 nM	95.4%	[6]
SAH	Plasma (500 µL)	D_4_-SAH			358.0 → 136.0 CE = 25 V	6 nM	6.5 nM	6.5–520 nM	90.2%	
SAM	Liver extract (5 µL)	D_3_-SAM	A: 0.1 % formic acid in H_2_O B: ACN + 0.1 % formic acid	Hypercarb column (3 µm, 2.1 × 30 mm)	399.3 → 250.3 CE = 25 V	N.D.	1250 nM	1250–320,000 nM	98.4%	[17]
SAH		^13^C_5_-SAH			385.3 → 136.3 CE = 27 V	N.D.	1250 nM	1250–320,000 nM	104.9%	
SAM	Plasma (200 µL)	D_3_-SAM	A: 10 mM ammonium formate B: acetonitrile	RP-Amide Supelco (3.5 µm, 3.0 × 150 mm)	399.0 → 250.1 CE = 32 eV	1 nM	8 nM	8–1024 nM	99.2–104.6%	[18]
SAH	Plasma (200 µL)	D_5_-SAH			385.1 → 136.2 CE = 28 eV	8 nM	16 nM	16–1024 nM	92.7–103.5%	
SAM	Plasma (20 µL)	D_3_-SAM	A: 4 mM ammonium acetate with 0.1% formic acid and 0.1% HFBA; B, methanol with 0.1% formic acid	Phenomenex EZ: faast, (4.0 µm, 250 × 2.0 mm)	399.0 → 250.0 N.D.	N.D.	12.5 nM	12.5–5000 nM	N.D.	[19]
SAH	Plasma (20 µL)	D_4_-SAH			385.0 → 136.0 N.D.	N.D.	12.5 nM	12.5–5000 nM	N.D.	
SAM	pooled homogenized embryos (ND)	D_3_-SAM	A: 100% methanol B: 4 mM ammonium acetate, 0.1% formic acid, 0.1% HFBA (pH 2.5)	Discovery HS F5; (5.0 µm, 50 × 2.1 mm)	399.2 → 136.1 CE = 26 V	10 nM	20 nM	20–25,000 nM	97%	[20]
SAH		D_3_-SAM			385.2 → 136.1 CE = 26 V	2.5 nM	10 nM	10–10,000 nM	92%	
SAM	Plasma (50 µL)	daptomycin	A: 5 mM ammonium formate (pH = 5.0), and B: ACN	Speed Core HILIC (2.6 µm, 100 × 2.1 mm)	385.1 → 88.0 CE = 44 V	5 nM	10 nM	10–5000 nM	64.20%	[7]
SAH	Plasma (50 µL)				399.1 → 250.1 CE = 14 V	1 nM	3 nM	3–5000 nM	62.53%	
SAM	Plasma (100 µL)	D_3_-SAM	proprietary aqueous/organic phases (Chromsystems)	analytical column; (Chromsystems)	399.0 → 250.0 CE= 20.4	1.005 nM	10 nM	10–200 nM	108%	[5]
SAH		D_4_-SAH			385.0 → 136.0 CE 22.9	0.081 nM	10 nM	10–200 nM	110%	
SAM	Plasma (50 µL)	D_3_-SAM	A: H_2_O with 0.5% formic acid and 0.25% HFBA B: ACN with 0.5% formic acid and 0.25% HFBA	Synergi Hydro-RP (4.0 µm, 150 × 3.0 mm)	399.0 → 250.0 CE = 14 eV	2.0 nM	3.0 nM	3–800 nM	90–108%	[21]
	CSF (50 µL)					2.0 nM	4.0 nM	4–800 nM	96–109%	
SAH	Plasma (50 µL)	D_4_-SAH			385.0 → 134.0 CE = 21 eV	0.1 nM	0.5 nM	8–1000 nM	90–108%	
	CSF (50 µL)					2.0 nM	4.0 nM	0.5–400 nM	96–109%	
SAM	IPECJ2 lysate (ND)	D_4_-HCY	A: 10 mM ammonium acetate B: 20% ACN in MeOH	XSelectHSS T3 (1.8 µm, 2.1 × 100 mm)	399.0 → 245.0 CE = 18 eV	ND	0.02 ng/10^6^ cells	2–500 ng/10^6^ cells	108.3–111.1%	[22]
	PIEC lysate (ND)								94.4–102.9%	
SAH	IPECJ2 lysate (ND)	D_4_-HCY			385.0 → 135.9 CE = 25 eV	ND	0.91 ng/10^6^ cells	1–100 ng/10^6^ cells	104.1–107.5%	
	PIEC lysate (ND)								101.1–114.5%	
SAM	Plasma (40 µL)	D_3_-SAM	A: 4 mM ammonium acetate with 0.1% formic acid and 0.1% HFBA; B: methanol with 0.1% formic acid	Phenomenex EZ: faast, (4.0 µm, 250 × 2.0 mm)	399.1 → 250.1 CE = 21 V	1 nM	12.5 nM	12.5–5000 nM	N.D.	[23]
SAH	Plasma (40 µL)	D_4_-SAH			385.1 → 136.1 CE = 24 V	1 nM	12.5 nM	12.5–5000 nM	N.D.	
SAM	Derivatized cell samples (180 µL)	warfarin	A: 0.1% acetic acid in H_2_O; B: 0.1% formic acid in ACN	XSelectHSS T3 (3.5 µm, 4.6 × 150 mm)	399.3 → 250.3 CE = 20 V	25.1	125.5	125.5–6276 nM	94.63–96.09%	
SAH					385.2 → 133.8 CE = 24 V	0.65	1.30	1.30–65.0 nM	96.90–106.75%	[24]
SAM	Placenta (100 mg)	N.D.	A: 0.1% formic acid in H_2_O; B: 0.1% formic acid in ACN	Synergi Fusion-RP (4.0 µm, 2.0 × 150 mm)	399.2 → 250.2 CE = 22 V	N.D.	N.D.	N.D.	N.D.	[25]
SAM	Plasma (40 µL)	D_3_-SAM	A: 5 mM HFBA; B: ACN	XSelectHSS T3 (2.5 µm, 2.1 × 100 mm)	399.0 → 250.0 CE = 14 eV	1 nM	3 nM	3–800 nM	98.8–103.0%	[26]
SAH	Plasma (40 µL)	D_4_-SAH			385.0 → 134.0 CE = 21 eV	0.5 nM	0.5 nM	0.5–400 nM	96.1–98.3%	

Abbreviations: ACN, acetonitrile; CE, collision energy; CSF, cerebrospinal fluid; FA, formic acid; HFBA, heptafluorobutyric acid; HILIC, hydrophilic interaction liquid chromatography; IS, internal standard; LC–MS/MS, liquid chromatography–tandem mass spectrometry; LOD, limit of detection; LLOQ, lower limit of quantification; N.D., not determined; PGC, porous graphitic carbon; RP, reversed phase; SAM, S-adenosylmethionine; SAH, S-adenosylhomocysteine. Values are reported as in the original publications. Where applicable, concentration units differ between studies. Linearity ranges originally reported in ng/mL were converted to nM using molecular weights of 398.44 g/mol for SAM and 384.40 g/mol for SAH.

The main challenge in determining SAM and SAH in biological matrices is the leakage of compound-free material. Therefore, choosing the right method to compensate for SAM/SAH as endogenous compounds is essential, even before starting sample preparation and assay optimization. If the endogenously presented compounds are analyzed, the distinction between analytes added during calibration and those typically presented in the matrix is not possible. The European Medicines Agency revised the International Harmonisation Committee’s recommendations (M10 document) and suggested potential bioanalytical solutions in section “Methods for analytes that are also endogenous molecules” [32]. The selected methods, along with comments specific to SAM and SAH determination, are outlined below.

(1) The surrogate matrix approach

The surrogate matrix approach is the easiest way to validate a method where an analyte-free matrix is not available. For example, as a surrogate for plasma, PBS or bovine albumin can be used. If the charcoal can remove the endogenous analytes of interest from the matrix, the special options of plasma are commercially available or can be prepared in-house in a laboratory. For example, in a study published by Bravo et al., water with formic acid was used as a surrogate matrix for method calibration [14]. It should be noted that when the surrogate matrix is used, the recovery differences (surrogate matrix vs. classic matrix) should be carefully evaluated.

(2) The surrogate analyte approach

In this method, a structurally similar analyte, i.e., an isotope-modified standard, is used for calibration [5,6,14,17,18,19,20,21,22,23,26]; however, in clinical samples, the original analyte is measured. The calibration curve constructed with the surrogate analyte is used to establish the target analyte concentration. Similar to the surrogate matrix approach, the evaluation of equality between surrogate analyte and classic analyte should be provided.

(3) Background subtraction

This method assumed use of a blank sample (without analyte addition for calibration) for systematic correction of analyte signals in calibrator by subtraction. For elimination of specific for LC-MS/MS interferences (matrix effect) caused, i.e., ion suppression, the values are compensated using analyte-to-internal standard ratio determined in the blank sample.

In most cases [5,7,18,19,20,21,22,26], gradient elution was employed for effective chromatographic separation on various stationary phases, including C8, C18, amide, and HILIC-based phases. Due to the hydrophobicity of the target compounds, the use of HILIC or amide phases is advantageous for enhancing the retention of SAM and SAH. For detection, the MRM (multiple-reaction monitoring) or SRM (single-reaction monitoring) method was utilized in positive electrospray ionization with apparatus-dependent collision energy ranging from 14 to 44 eV [5,6,7,14,17,18,19,20,21,22,23,24,25,26].

The sensitivity of developed methods depended on the utilized matrix and the individual sensitivity of the mass spectrometer. The most sensitive method set the lower limit of detection (LOD) and lower limit of quantification (LLOQ) at 1 nM and 3 nM, respectively. The recovery of analytes established in the analyzed literature ranged from 50 to 110%, with or without compensation using the internal standard [5,6,7,14,17,18,19,20,21,22,23,24,25,26]. The recovery values reported for SAM and SAH varied substantially across studies, with some methods reporting recoveries of approximately 50%. In assays employing stable isotope-labelled internal standards, such recovery levels were considered acceptable in the original validations, as extraction losses were consistently compensated during quantification. In this context, recovery should not be interpreted as a standalone measure of analytical robustness, if accuracy and precision acceptance criteria were fulfilled. However, differences in recovery may impact inter-study comparability when internal standardization strategies, extraction protocols, or matrix compositions differ. Methods relying on non-isotopic or single internal standards may be more susceptible to variability introduced by incomplete recovery. Therefore, recovery values must be interpreted in conjunction with the overall validation strategy, particularly for endogenous metabolites such as SAM and SAH.

The analytical scope of the reviewed methods varied considerably between studies. In several publications, SAM and SAH were quantified as the sole target analytes using dedicated assays optimized for clinical or translational applications [17,18,19,20,23,25]. In contrast, other studies employed targeted metabolomics platforms in which SAM and SAH were measured in parallel with additional metabolites of the methionine or one-carbon cycle, such as homocysteine, methionine, cystathionine, or related cofactors [5,6,7,14,21,22,24,26]. These methodological differences influence assay design, validation depth, and analytical performance. Dedicated SAM/SAH assays typically prioritize absolute quantification and matrix-specific validation, whereas multi-analyte approaches emphasize broader pathway coverage and throughput. Consequently, the direct comparison of validation parameters between these two analytical strategies should be interpreted with caution.

The chromatographic separation of SAM and SAH is particularly challenging due to their high polarity and close structural similarity. The key differentiating feature is the permanently positively charged sulfonium group of SAM, which influences its retention and ionization behavior compared with the neutral thioether structure of SAH. This physicochemical difference is exploited in LC–MS/MS methods through the use of hydrophilic interaction liquid chromatography (HILIC), amide-based stationary phases, porous graphitic carbon, or polar-embedded reversed-phase columns, typically operated under acidic conditions.

In clinical laboratory practice, reliable discrimination between SAM and SAH does not rely solely on chromatographic resolution but rather on a combination of optimized chromatography, compound-specific multiple-reaction monitoring transitions, and stable isotope dilution. The use of isotope-labelled internal standards corrects for matrix effects and ensures analytical selectivity and robustness, making LC–MS/MS the reference method for routine clinical determination of SAM and SAH.

## 5. Analytical Phase—Other Methods for SAM and SAH Determination

Not only LC-MS/MS has been applied for the determination of SAM and SAH; immunoassay-based approaches have also been explored as potential alternatives, particularly in settings where access to advanced mass spectrometry is limited. However, their application is constrained by fundamental limitations in specificity and sensitivity. Hao et al. demonstrated that no conventional antibody directed against free SAM is available, and that antibodies developed against SAH are not suitable for direct use in whole blood or plasma matrices [33]. To overcome these limitations, the authors developed a competitive immunoassay using synthetic haptens and optimized assay conditions, rather than a classical sandwich ELISA format. This approach enabled the indirect quantification of SAM and SAH, achieving detection limits of approximately 2 nM for SAM and 15 nM for SAH, with reported affinity constants of 7.29 × 10^10^ L/mol and 2.79 × 10^8^ L/mol, respectively [33]. Although the method demonstrated utility in epidemiological and exploratory studies—allowing assessment of sex-dependent differences and disease-related alterations in SAM/SAH homeostasis—it remains limited by matrix compatibility and analytical robustness when compared with LC-MS/MS. Overall, while immunoassay-based approaches such as ELISA may provide a technically simpler and more accessible option for exploratory or epidemiological studies, LC-MS/MS remains the method of choice for SAM and SAH quantification due to its superior specificity, sensitivity, and robustness in complex biological matrices.

## 6. Alterations in SAM and SAH Concentrations and the SAM/SAH Ratio Across Disease Conditions

Alterations in the concentrations of S-adenosylmethionine (SAM) and S-adenosylhomocysteine (SAH), as well as in the SAM/SAH ratio, have been increasingly reported across a wide range of disease conditions (Table 2). These changes reflect disruptions in cellular methylation capacity and one-carbon metabolism and are often associated with disease development, progression, and severity. In a study of 81–291 serum samples, the average SAM and SAH concentrations in healthy individuals were 386 ± 216 nM and 257 ± 151 nM, respectively, with a methylation index (MI) of 2.2 ± 1.9. The SAM levels were higher in women (~28% higher) and declined with age. SAM was markedly reduced (<120 nM) in liver diseases, accompanied by a low MI (<0.5), while SAH changes were less pronounced [33]. Certain cancers have shown alterations in both SAM and SAH, with wide MI variability, suggesting that these metabolites and the MI may serve as potential biomarkers of disease severity and health status [33]. Plasma concentrations of SAM are significantly elevated in patients with lung cancer and may prove useful for the diagnosis of early lung cancer, in combination with chest CT (CT—computed tomography) [34].

SAM and SAH concentrations have been implicated in the pathogenesis of MASLD-HCC (MASLD—metabolic dysfunction-associated steatotic liver disease, HCC—hepatocellular carcinoma). Specifically, in a pilot case-control study of 69 patients with MASLD-HCC and 136 controls, the plasma SAM levels (mean 121 vs. 96 nmol/L) and SAM/SAH ratios (2.09 vs. 1.48) were significantly higher in MASLD-HCC cases. Multivariable-adjusted analyses showed that elevated SAM levels (OR 2.76, 95% CI 1.38–5.72) and higher SAM/SAH ratios (OR = 2.30, 95% CI 1.15–4.73) were associated with increased odds of MASLD-HCC, whereas SAH alone was also linked to disease. These findings suggest that plasma SAM and the SAM/SAH ratio may serve as noninvasive markers for HCC risk in MASLD patients [35].

In addition, alternations in SAM and SAH concentrations are also associated with renal dysfunctions. For instance, in a prospective study, plasma homocysteine and related metabolites were measured in 25 patients on regular hemodialysis and 40 healthy volunteers. The patients exhibited markedly elevated plasma homocysteine (36.6 ± 3.6 µmol/L), SAM (381 ± 32 nmol/L), and SAH (1074 ± 55 nmol/L) compared to controls (homocysteine 6.8 ± 0.4 µmol/L, SAM 60 ± 3 nmol/L, SAH 24.4 ± 1.1 nmol/L). The SAM/SAH ratio was significantly reduced in patients (0.36 ± 0.02 vs. 2.7 ± 0.2 in controls), reflecting impaired methylation capacity in end-stage renal disease [36]. Similarly, in a case-control study including 50 chronic kidney disease (CKD) patients and 20 healthy volunteers, urinary SAM levels and SAM/SAH ratios were significantly lower in CKD patients (*p* < 0.001 and *p* = 0.01, respectively). Plasma levels of SAM, SAH, and homocysteine were elevated in CKD, whereas the plasma SAM/SAH ratio was significantly decreased. Urinary SAM and the SAM/SAH ratio were strongly correlated with the estimated glomerular filtration rate. These findings indicate that CKD is associated with reduced urinary SAM and the SAM/SAH ratio, and that both measurements may serve as promising noninvasive indicators of renal dysfunction [37]. Moreover, in a pilot clinical study, SAM and SAH were quantified in EDTA plasma from 16 de novo kidney transplant patients using a validated LC-MS/MS assay, with eight healthy controls. Plasma SAM and SAH concentrations were significantly elevated in patients preceding acute rejection or nephrotoxicity compared with controls and transplant patients without allograft dysfunction, whereas methylation potentials—SAM/SAH ratio (SAR), creatinine-adjusted ratio (CAR), and erythrocyte-adjusted ratio (EAR)—were significantly lower in patients (1.48 ± 0.34, 1.21 ± 0.94, 1.38 ± 0.23, respectively) compared with healthy controls (SAR 3.23 ± 0.33). These findings suggest that SAM and SAH may serve as early molecular markers for transplant complications, and that altered SAM/SAH levels and reduced methylation potential may provide additional insight into allograft dysfunction [18].

Recent studies propose that SAH, and not Hcy, plays a role in cardiovascular disease (CVD) and might serve as a novel biomarker for the early clinical identification of this disorder. In a study of 160 participants (aged 40–80 years) with chest pain and suspected CAD, the plasma SAM and SAH levels were measured, and coronary artery lesions were assessed by angiography. The SAH levels were significantly higher in the CAD group (23.09 ± 2.4 nmol/L) compared with the atherosclerosis (AS) group (19.2 ± 1.5 nmol/L), while the SAM/SAH ratios were lower in CAD (4.1 ± 1.1 vs. 5.1 ± 0.7). The SAM, homocysteine, folate, and vitamin B12 levels were similar between groups. Coronary artery lesions were independently associated with SAH (β = 11.8, 95% CI 5.88–17.7, *p* < 0.05), suggesting that plasma SAH might be used to identify cardiovascular disease at an early stage. [38]. In the context of cardiovascular conditions, patients with peripheral arterial occlusive disease (PAOD) also often exhibit altered homocysteine metabolism, which is associated with elevated plasma and erythrocyte levels of SAH and changes in SAM. This results in a decreased SAM/SAH ratio, suggesting impaired methylation capacity in these individuals. In a study of 61 patients with PAOD (aged 49–93) and 50 healthy controls (aged 41–87), plasma homocysteine (15.5 vs. 10.4 µmol/L), and plasma SAM (107 vs. 52.3 nmol/L) and SAH (55.0 vs. 23.1 nmol/L) were significantly elevated in patients, while the SAM/SAH ratio was decreased (1.92 vs. 2.52). In erythrocytes, patients showed higher SAH (309 vs. 205 nmol/L) and lower SAM (3351 vs. 3732 nmol/L) and SAM/SAH ratio (11.8 vs. 19.1). Hematocrit levels were also reduced in patients (0.35 vs. 0.42 L/L) and correlated with the erythrocyte SAM/SAH ratio [39].

Alterations in one-carbon metabolism, particularly elevated concentrations of SAH and changes in SAM, have been observed in Alzheimer’s disease (AD). These metabolic disturbances suggest a link between disrupted methylation capacity and phospholipid metabolism, which may contribute to cerebrovascular dysfunction and neurodegenerative processes characteristic of AD [40]. In a single-center, cross-sectional study of 60 Alzheimer’s disease (AD) patients and 60 controls, CSF levels of SAM, SAH, and homocysteine were measured alongside plasma vitamin B_12_, folate, and APOE genotype. AD patients had significantly lower CSF SAM levels (193 ± 31 vs. 207 ± 37 nmol/L; *p* = 0.032) and lower SAM/SAH ratio (7.6 ± 2.4 vs. 9.1 ± 2.8; *p* = 0.003). Exploratory analysis showed that APOE4 carriers had lower CSF SAM levels (189 ± 30 vs. 207 ± 36 nmol/L; *p* = 0.010), with 63% of individuals in the lowest CSF SAM quartile carrying APOE4 compared with 17% in the highest quartile. These findings indicate that AD is associated with reduced CSF SAM, partly related to APOE4 genotype [9]. On the other hand, in a study of 26 Alzheimer’s disease patients and 29 healthy controls, plasma SAH, SAM, and homocysteine levels were measured alongside erythrocyte phosphatidylcholine (PC) and phosphatidylethanolamine (PE) composition and their polyunsaturated fatty acids. AD patients had significantly higher plasma SAH, SAM, and homocysteine levels (all *p* < 0.001). Plasma SAH positively correlated with homocysteine (r = 0.738, *p* < 0.001) [40]. SAM and SAH concentrations were also measured in postmortem brains of 11 Alzheimer’s disease patients and 14 matched controls. SAM levels were decreased by 67–85% and SAH by 56–79% across cerebral cortex, hippocampus, and putamen. Reduced SAM may result from excessive utilization in polyamine biosynthesis, potentially compromising brain metabolism and function, and may explain cognitive improvements observed in some AD patients following SAM therapy [41]. Beyond its impact on Alzheimer’s disease, altered SAM and SAH metabolism may be also associated with the pathophysiology of dementia, highlighting a role of disrupted methylation in neurodegenerative disorders. For example, in a prospective cohort of 1371 older Japanese adults, higher serum SAM levels and SAM/SAH ratios were associated with a lower risk of dementia and death, whereas higher SAH levels were linked to increased risk. During a median follow-up of 10.2 years, multivariable-adjusted analyses showed a significant trend across quartiles for each metabolite, suggesting that the balance of methionine metabolites may influence dementia and mortality risk [42]. In the context of neurodegenerative disorders, significant differences in SAM and SAH levels have also been observed in Parkinson’s disease. In a study of 20 parkinsonian patients and 12 healthy controls, whole-blood SAM levels were significantly lower in patients (383.1 ± 41.5 nM) compared with controls (680.6 ± 30.9 nM), whereas the SAH levels did not differ between groups. These findings indicate altered SAM metabolism in Parkinson’s disease [43]. Beyond classical neurodegenerative disorders, alterations in SAM and SAH levels have also been reported in neurodevelopmental conditions such as autism spectrum disorder. In a study of 59 children with autism spectrum disorder (ASD) and 40 neurotypical controls, plasma SAM levels and the SAM/SAH ratio were significantly elevated in ASD (SAM 83.2 ± 13.7 nmol/L vs. 66.2 ± 14.9 nmol/L, SAM/SAH 3.87 ± 0.93 vs. 2.00 ± 0.76), whereas the SAH levels were significantly lower (median 20.8 nmol/L, IQR 18.1–24.5 vs. 37.9 nmol/L, IQR 29.2–40.8, *p* < 0.001), and several other one-carbon metabolism metabolites were also decreased. These findings indicate disturbances in methionine methylation and trans-sulfuration pathways in ASD and suggest a potential role of OCM (one-carbon metabolism) as therapeutic target [44]. Furthermore, a meta-analysis of 22 studies compared peripheral blood levels of methionine (Met), SAM, SAH, and the SAM/SAH ratio in individuals with autism spectrum disorder and controls. ASD was associated with significantly decreased Met (Hedges’ g = −0.62), SAM (g = −0.60) and SAM/SAH ratio (g = −0.98) and increased SAH (g = 0.69). These findings indicate that ASD is linked to impaired methylation capacity, reflected in altered SAM, SAH, and SAM/SAH levels [45].

**Table 2:** Reported concentrations of SAM, SAH, and the SAM/SAH ratio across disease conditions.

Condition	Matrix	SAM	SAH	SAM/SAH Ratio	Direction vs. Controls	Key Observation	References
Healthy controls	serum/plasma	386 ± 216 nM	257 ± 151 nM	2.2 ± 1.9	-	wide physiological variability	[33]
Liver disease	serum	<120 nM	slight change	<0.5	↓ SAM, ↓ ratio	markedly reduced methylation index	[33]
MASLD-HCC	plasma	121 vs. 96 nM	↑	2.09 vs. 1.48	↑ SAM, ↑ ratio	associated with increased HCC risk	[35]
Lung cancer	plasma	↑	-	-	↑ SAM	potential early diagnostic marker	[34]
Hemodialysis	plasma	381 ± 32 nM	1074 ± 55 nM	0.36 ± 0.02	↑ SAH, ↓ ratio	severely impaired methylation capacity	[36]
CKD	plasma/urine	↑ plasma ↓ urine	↑	↓	↓ ratio	correlates with eGFR	[37]
Kidney transplant rejection	plasma	↑	↑	1.48 vs. 3.23	↓ ratio	early marker of allograft dysfunction	[18]
CAD	plasma	unchanged	23.1 vs. 19.2 nM	4.1 vs. 5.1	↑ SAH, ↓ ratio	SAH independently associated with lesions	[38]
PAOD	plasma	107 vs. 52 nM	55 vs. 23 nM	1.92 vs. 2.52	↑ SAM, ↑ SAH, ↓ ratio	impaired erythrocyte methylation	[39]
Alzheimer’s disease	CSF	193 vs. 207 nM	-	7.6 vs. 9.1	↓ ratio	linked to APOE4 genotype	[9]
	brain tissue	↓ 67–85%	↓ 56–79%	-	Strong ↓ SAM	suggests excessive utilization	[41]
Dementia (prospective cohort study)	serum	↑ protective	↑ harmful	↑ protective	Direction-dependent	high ratio associated with lower risk	[42]
Parkinson’s disease	whole blood	383 vs. 681 nM	No change	-	↓ SAM	altered SAM metabolism	[43]
ASD	plasma	83 vs. 66 nM	↓	3.87 vs. 2.00	↑ ratio	altered methylation/transsulfuration	[44]
ASD (meta-analysis)	blood	↓	↑	↓	↓ ratio	impaired methylation capacity	[45]

ASD, autism spectrum disorder; CAD, coronary artery disease; CKD, chronic kidney disease; CSF, cerebrospinal fluid; HCC, hepatocellular carcinoma; MASLD, metabolic dysfunction-associated steatotic liver disease; PAOD, peripheral arterial occlusive disease; SAM, S-(Adenosyl)methionine; SAH, S-(Adenosyl)homocysteine; SAM/SAH ratio, methylation index.

## 7. Looking for Reference Ranges

The SAM and SAH tests are commercially available in hospital laboratories. Well-defined reference ranges for SAM, SAH, and the SAM/SAH ratio are essential for the accurate interpretation of deviations observed in pathological conditions. Published data on SAM and SAH levels, as well as the SAM/SAH ratio, in healthy individuals are summarized in Table 3 and are based on analyses of human serum, plasma, erythrocytes, and whole blood.

Data are reported as mean ± standard deviation, median with interquartile range (IQR), or geometric mean (GM), depending on the original publication. Reported plasma SAM concentrations in healthy patients were approximately 52–148 nmol/L, while plasma SAH concentrations ranged from approximately 15–128 nmol/L. The SAM/SAH ratio varied between approximately 1.5 and 10.0, depending on the biological matrix and analytical method. Considerable interstudy variability reflects differences in sample type, sample handling, measurement techniques, analytical methodology, population characteristics, and data presentation.

## 8. Discussion

In accordance with the current guidelines for transparent and reproducible evidence synthesis reporting [13], the analytical studies listed in Table 1 were examined. The review focused on their methodological goals, validation levels, and matrix specificity. Additionally, each study was explicitly critiqued for its analytical strengths and weaknesses to prevent unfair comparisons between assays designed for different purposes and biological matrices.

Throughout the development of the methods [5,7,14,17,19,20,21,23,26] all validation parameters—such as detection and quantification limits, calibration models, and recovery or accuracy measures—were documented. This offers both methodological guidance for SAM and SAH quantification using LC-MS/MS and provides analytical proof of concept. In contrast, studies such as [25,27,28] utilized previously published, well-characterized, and validated LC-MS/MS assays to explore epidemiological or experimental questions. These studies did not re-validate the analytical performance for the new samples and matrices; instead, according to reporting guidelines, they should be viewed as applications of methods whose performance was already established in the cited development studies. Therefore, reassigning or extrapolating these validation parameters in this context would be methodologically inappropriate.

Multiple studies, including [5,18,19,21,23,26], showcase the growing potential of stable isotope dilution LC-MS/MS analysis. These works emphasize that isotopically labelled SAM and SAH analogues enhance detection limits—reaching low nanomolar levels—and calibration ranges, while addressing matrix-dependent ionization suppression. This method is regarded as the gold standard for quantifying SAM and SAH in plasma and cerebrospinal fluid (CSF). In contrast, certain targeted metabolomic and cellular assays [22,24] depend on a single or non-isotopic internal standard to enable the coverage of multiple analytes. Although these methods may be suitable for pathway investigation, they are less ideal for absolute quantification or direct comparison with isotope-dilution techniques. This difference indicates that the analytical performance should be evaluated considering the biological and experimental context in which they are employed.

The high polarity and zwitterionic nature of SAM and SAH have historically made chromatography challenging, necessitating the use of ion-pairing reagents (a feature of the early methods of Burren, Gardner, and others). These reagents are still used for this purpose but can contaminate and degrade the MS source. Subsequent studies have investigated other stationary phases, such as porous graphitic carbon [17] or polar-embedded reversed-phase columns [18,21], which improve retention while reducing the need for ion-pairing. A key finding of Table 1 is that no single chromatographic format is optimal for all analyses. Matrix effects are substantial: plasma, cerebrospinal fluid, milk, tissue homogenates, and cell lysates each impose distinct physicochemical and biochemical constraints. Hence, validation parameters must be considered in the context of the matrix in which the assay was developed, rather than any biological entity. Despite methodological advances, the lack of standardization leads to variations in calibration ranges, limits of quantification, and concentration units. Plasma assays typically measure molar concentrations (nM), while cell-based assays are often normalized to cell count or protein concentration. Although these methods are mathematically equivalent, such differences complicate comparisons of data across studies. Therefore, in accordance with systematic review reporting guidelines, Table 1 presents the methods as originally published. This approach aims to prevent mischaracterization of methods and underscores the absence of standardized reporting of intermediates in one-carbon metabolism.

Overall, the literature indicates that LC-MS/MS is a reliable and adaptable method, particularly effective for measuring SAM and SAH in plasma and other biofluids under stable-isotope dilution conditions. Comparing results between laboratories is valid only if the same internal standards, chromatography systems, matrix effects, and validation protocols are used, or if reporting standards are aligned. Otherwise, there is a risk of confusing exploratory metabolomics with targeted pathway analysis and clinical bioanalysis, which recent guidance explicitly warns against. By combining this methodological review with honest evaluations of each approach’s strengths and weaknesses, we can better interpret SAM and SAH data from both research and clinical studies, adhering to best practices for transparency and reproducibility. Additionally, matrix-specific validation should become standard for LC-MS/MS methods in new biological matrices. Applying pre-validated methods to new samples, such as milk, placenta, or tissue homogenates, requires rechecking matrix effects, recoveries, and detection limits. Even minimal validation data greatly enhances cross-study comparability. The research community, as well as validation recommendations, should prioritize stable-isotope dilution techniques for the absolute quantification of SAM and SAH in future studies. Although using a single internal standard can be acceptable in exploratory or pathway-focused metabolomics, understanding and reporting its limitations is crucial. Cost-effective, widely available isotopically labelled standards will support method harmonization. Furthermore, ion-pairing reagents should be used sparingly. Although effective for retaining polar sulfonium species, alternative chromatographic methods—such as advanced polar-embedded reversed-phase columns, mixed-mode columns, or improved HILIC—offer greater instrument robustness and long-term transferability without sacrificing sensitivity. The field should also emphasize standardized reporting of analytical performance parameters, including calibration ranges, detection and quantification limits, and normalization methods. Adopting such standards in targeted metabolomics and one-carbon metabolism research will enhance reproducibility and transparency. Lastly, researchers need to carefully consider the intended application of SAM and SAH measurements—whether for clinical quantification, nutritional analysis, mechanistic insights, or exploratory studies—to prevent misinterpretation, misrepresentation, and issues with translating and comparing methodologies. In summary, these recommendations aim to improve the robustness, transparency, and comparability of SAM and SAH analytical results across clinical, nutritional, and experimental research, amid ongoing advancements in analytical techniques.

Matrix effects are a major source of variability in LC–MS/MS analysis of SAM and SAH, particularly because of their endogenous nature and high polarity. Quantitative estimates of ion suppression or enhancement were not consistently reported across the reviewed studies, limiting direct comparison of matrix effect values. However, most clinically oriented assays use stable-isotope-labelled internal standards, which effectively compensate for matrix-dependent ionization effects. In contrast, methods relying on non-isotopic or single internal standards may be more susceptible to residual matrix effects, which should be considered when comparing analytical performance across studies [5,6,7,14,17,18,19,20,21,22,23,24,25,26].

In several studies, the reported lower limit of quantification does not align with the lower boundary of the calibration range. This reflects differences in validation strategy, as LLOQ was often defined conservatively based on accuracy and precision criteria, whereas broader calibration ranges were established to cover physiologically relevant concentrations. Such discrepancies reflect methodological choices rather than analytical inconsistency. Previous studies have addressed analytical aspects of SAM and SAH determination, including immunoassay-based approaches and LC–MS/MS method development and validation. However, these publications have typically focused on individual analytical techniques, specific biological matrices, or selected clinical applications [5,6,7,14,17,18,19,20,21,22,23,24,25,26]. The present review provides an integrated comparison of validated analytical methods for SAM and SAH quantification across different biological matrices, with particular emphasis on validation parameters, matrix effects, analyte stability, and clinical applicability. By linking methodological performance with reported concentration ranges in healthy individuals and patient populations, this review aims to clarify current limitations and support the standardized interpretation of SAM and SAH measurements in both research and clinical laboratory settings.

In clinical laboratory practice, SAM and SAH are predominantly determined by liquid chromatography–tandem mass spectrometry (LC–MS/MS). These assays are implemented as validated in-house methods and typically employ stable-isotope dilution and targeted quantification, providing the specificity and sensitivity required for low-nanomolar endogenous concentrations. In contrast, immunoassay-based methods for the determination of SAM and SAH are not routinely used in clinical diagnostics. Although such approaches have been explored for research purposes, their clinical application remains limited due to constraints on specificity and matrix interference. Consequently, LC–MS/MS currently serves as the reference analytical technique for the measurement of SAM and SAH in clinical laboratory practice. Therefore, the interpretation of clinically reported SAM and SAH concentrations should always be considered in the context of LC–MS/MS-based methodologies and matrix-specific validation.

## 9. Conclusions

The accurate measurement of S-adenosylmethionine (SAM) and S-adenosylhomocysteine (SAH) is crucial due to their significant roles in cellular methylation processes and their utility as biomarkers in various disease conditions. The SAM/SAH ratio is widely recognized as a key indicator of methylation capacity and epigenetic status. This review highlights the growing need for reliable, sensitive, and precise analytical techniques to measure SAM and SAH in biological samples. Advances in chromatographic and mass spectrometric methods have significantly improved the accuracy and efficiency of these analyses. However, challenges such as matrix effects, analyte stability, and consistency between laboratories still need to be addressed. Due to the clinical importance of these metabolites in disease advancement, diagnosis, and treatment assessment, ongoing improvement and verification of analytical techniques are necessary. Upcoming initiatives should focus on standardizing protocols, enhancing sample preparation techniques, and expanding the implementation of high-throughput and multiplexed assays for broader clinical and research applications.

## Figures and Tables

**Table 3:** Examples of proposed reference values of SAM, SAM, and the methylation index in healthy individuals are presented in the literature.

Source	SAM [nM]	SAH [nM]	SAM/SAH Ratio	Ref.
plasma	120.6 ± 18.1	21.5 ± 3.2	5.6 ± 1.0	[14]
urine	22,100 ± 11,000	2200 ± 1000	10.0 ± 1.9	[14]
plasma	53.1 ± 21.6	15.2 ± 0.66	2.1	[6]
plasma	148 ± 62	128 ± 72	N.D.	[7]
plasma	88.5 ± 18.1	25.7 ± 9.9	N.D.	[21]
CSF	191.4 ± 31.1	14.3 ± 2.5	N.D.	[21]
plasma	63.3 (54.9–75.9)	26.5 (95% CI: 25.2–28.0)	N.D.	[28]
adult plasma	59 (IQR 16)	26 (IQR 8)	N.D.	[27]
maternal plasma	42 (IQR 16)	41 (IQR 14)	N.D.	[27]
cord plasma	56 (IQR 86)	43 (IQR 39)	N.D.	[27]
breast milk	1830 (IQR 805)	263 (IQR 192)	N.D.	[27]
CSF	154 (IQR 29)	10 (IQR 6)	N.D.	[27]
serum	60.71 (46.40–88.71)	18.96 (16.19–23.48)	3.07 (2.33–4.53)	[42]
serum	386 ± 216.2	256.9 ± 150.7	2.2 ± 1.9	[33]
plasma	96 ± 43	65 ± 22	1.48 ± 0.47	[35]
plasma	60 ± 3	24.4 ± 1.1	2.7 ± 0.2	[36]
plasma	52.3	23.1	2.52	[39]
erythrocytes	3732	205	19.1	[39]
whole blood	680.6 ± 30.9	N.D.	N.D.	[43]
plasma	66.2 ± 14.9	37.9 (29.2–40.8)	2.00 ± 0.76	[44]
plasma	95.2 ± 21.6	30.4 ± 6.2	3.23 ± 0.33	[18]

CSF, cerebrospinal fluid; IQR, interquartile range; N.D., not determined; Ref., references; SAM, S-(Adenosyl)methionine; SAH, S-(Adenosyl)homocysteine; SAM/SAH, SAM/SAH ratio, methylation index.

## Data Availability

No new data were created.

## Abbreviations

The following abbreviations are used in this manuscript:

ACN	Acetonitrile
AD	Alzheimer’s disease
AdoHcy	Adenosylhomocysteine
AdoMet	Adenosylmethionine
APOE	Apolipoprotein E
APOE4	Apolipoprotein E4
AS	Atherosclerosis
ASD	Autism spectrum disorder
CAD	Coronary artery disease
CAR	Creatinine-adjusted ratio
CI	Confidence interval
CKD	Chronic kidney disease
CSF	Cerebrospinal fluid
CT	Computed tomography
DTT	Dithiothreitol
EAR	Erythrocyte-associated ratio
EDTA	Ethylenediaminetetraacetic acid
ELISA	Enzyme-Linked Immunosorbent Assay
FA	Formic acid
HCC	Hepatocellular carcinoma
HFBA	Heptafluorobutyric acid
HILIC	Hydrophilic interaction liquid chromatography
IA	Immunoassay
IQR	Interquartile range
LC-MS/MS	Liquid chromatography–tandem mass spectrometry
LOD	Limit of detection
LLOQ	Lower limit of quantification
MASLD	Metabolic dysfunction-associated steatotic liver disease
Met	Methionine
MI	Methylation index
N.D.	Not determined
OCM	One-carbon metabolism
PAOD	Peripheral arterial occlusive disease
PBS	Phosphate-buffered saline
PC	Phosphatidylcholine
PE	Phosphatidylethanolamine
PGC	Porous graphitic carbon
RP	Reversed phase
SAH	S-adenosylhomocysteine
SAM	S-adenosylmethionine
SAM/SAH	SAM to SAH ratio
SD	Standard deviation
SPE	Solid-phase extraction
TCA	Trichloroacetic acid
nM	Nanomolar
µM	Micromolar
